# A new insight into the fabrication of colloidal isotropic ZnO nanocrystals by an organometallic approach†

**DOI:** 10.1039/d4na00933a

**Published:** 2025-03-12

**Authors:** Anna Wojewódzka, Małgorzata Wolska-Pietkiewicz, Roman H. Szczepanowski, Maria Jędrzejewska, Karolina Zelga, Janusz Lewiński

**Affiliations:** a Faculty of Chemistry, Warsaw University of Technology Noakowskiego 3 00-664 Warsaw Poland malgorzata.pietkiewicz@pw.edu.pl janusz.lewinski@pw.edu.pl; b International Institute of Molecular and Cell Biology Ks. Trojdena Street 4 02-109 Warsaw Poland; c Institute of Physical Chemistry, Polish Academy of Sciences Kasprzaka 44/52 01-224 Warsaw Poland

## Abstract

The study of factors controlling nanocrystal (NC) growth is essential for uncovering and understanding nanomaterial formation, which typically involves a complex sequence of precursor reactions, nucleation, and growth processes. Herein, as part of the continuous development of the self-supporting organometallic approach for the preparation of quantum-sized colloidal zinc oxide (ZnO) NCs, we selected a series of [EtZn(X)]-type carboxylate precursors, where X = methoxyacetate, 2-(2-methoxyethoxy)acetate, or 2-[2-(2-methoxyethoxy)ethoxy]acetate, as model self-supporting systems with varying carboxylate tail lengths. The controlled exposure of a [EtZn(X)]-type precursor solution to air afforded colloidal ZnO NCs with a narrow unimodal size distribution and coated with strongly anchored X-type ligands. Employing optical spectroscopy techniques, we investigate how the growth dynamics of NCs depend on the length of the carboxylate tail. Moreover, leveraging analytical ultracentrifugation (AUC), we meticulously examined the behavior of NCs in solution under centrifugal forces to gain valuable insights into their stability and aggregation tendencies. This study not only enhances understanding of the underlying ‘living growth’ of organometallic-derived nanostructures that leads to the formation of thermodynamically stable and monodispersed ZnO NCs but also significantly contributes to the ongoing development of more effective methods for synthesizing colloidal ZnO NCs, thereby advancing the field of materials science.

## Abstract 2

In this study, we aimed to elucidate the “living growth” mechanism of ZnO nanocrystals (NCs) and employed analytical ultracentrifugation to assess their stability and aggregation tendencies.

## Graphical abstract

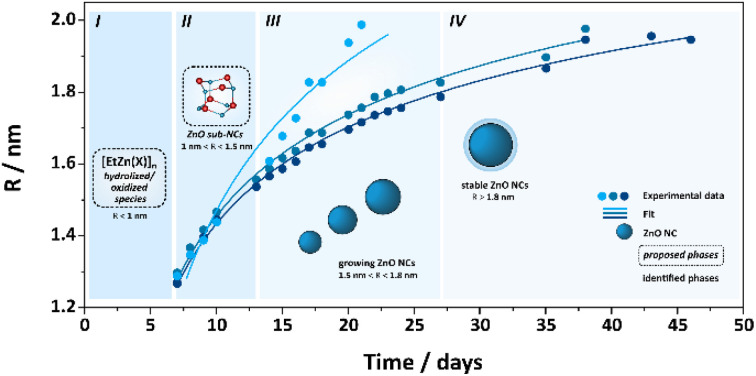

## Introduction

Colloidal zinc oxide nanocrystals (ZnO NCs) are of widespread importance for their unique combination of physicochemical properties and their wide spectrum of technological applications.^1–5^ Unquestionably, in the recent three decades, the wet inorganic sol–gel technique has been a ubiquitous method for the preparation of ZnO NCs.^4,6,7^ A typical sol–gel process involves the conversion of a pre-heated alcoholic zinc salt solution into a transparent sol, followed by the addition of alkali metal hydroxide. While this simple process affords diverse nanostructured materials,^6–10^ it has several serious limitations. Notably, multi-stage transformations take place at the early stages of the process (*t* < 5 min),^8,10–12^ and the resulting NCs in the quantum confinement regime (*d* < 10 nm) exhibit a high density of core-type and surface-type defects (including inherently incorporated alkali metal ions^13^), that manifest themselves mainly *via* inorganic–organic interface instability^14^ with a strong tendency to asymmetrical growth and aggregation over time. The addition of a surfactant is usually required to achieve the long-term stability of the resulting dispersions. Imperfections, such as a highly defective surface, instability of the interface, and the lack of accurate control and reproducibility over the synthesis, preclude using sol–gel-derived ZnO NCs for large-scale applications.

An advantageous alternative to the omnipresent inorganic sol–gel methods are wet-organometallic approaches. Chaudret and co-workers developed a low-temperature surfactant-assisted organometallic procedure based on a homoleptic organozinc precursor affording colloidal ZnO NCs coated with neutral L-type ligands,^15–17^ and recently this type of approach was developed further by our group towards ligand-free and easily dispersible bare quantum-sized crystals.^18,19^ Another breakthrough in the preparation of high-quality ZnO NCs has been achieved through the development of a general one-pot self-supporting organometallic (OSSOM) method involving the controlled exposition of a [EtZn(X)]-type precursor solution to air at ambient temperature (X = monoanionic organic ligand, *i.e.*, carboxylate,^20–24^ phosphinate,^25^ phosphate,^14^ aminoalcoholate^26^ and benzamidinate^27^), which leads to an exquisite variety of quantum-sized ZnO crystals coated with anchored X-type ligands. Systematic studies demonstrated that the character of the supporting ligand X is a dominant factor controlling the size, morphology and surface chemistry of the resulting NCs exhibiting a narrow unimodal size distribution. For example, the application of a series of organozinc complexes incorporating aminoalkoxide ligands provided NCs with sizes in the range of 1.4 nm to 8 nm determined only by changing the skeleton of the supporting ligand.^26,28^ The superiority of the OSSOM derived ZnO NCs over those prepared by the sol–gel process was nicely demonstrated by advanced comparative studies through dynamic nuclear polarization (DNP-)enhanced solid-state nuclear magnetic resonance.^14^ Moreover, the OSSOM provides bio-friendly ZnO NCs well-protected by a densely-packed ‘impermeable’ organic shell,^20–22^ which is prone to further processability.^24^ The unprecedented high-quality of the resulting NCs is also well-documented by the observed ultra-long-lived electron–hole separation (up to 2.2 μs)^29^ and the negligible nano-specific toxicity,^20,21^ which likely results from the unique NC-ligand interface.

Although the study of NC growth is essential to uncovering and understanding the mechanism of nanomaterial formation, it is also a challenging issue.^30–32^ Noticeably, several studies have investigated the growth kinetics of isotropic ZnO NCs using conventional sol–gel procedures.^9,12,33–35^ In turn, the solution organometallic approach has been investigated only in the context of anisotropic growth systems^36,37^ mediated by long-chain amines as L-type ligands, *e.g.*, primary amines promote zinc-amido oligomer formation, leading to the growth of ZnO nanorods, while secondary amines, due to steric hindrance, limit this process. Thus, differences in the morphology arise from ligand dynamics at the NC surface.^36^ Kahn *et al.* also demonstrated that ZnO NC growth follows an oriented attachment process, strongly influenced by the water concentration, hydrolysis rate, mixing time, and the coating ligand chain length.^37^ Recently, we provided an in-depth understanding of the mechanisms of ZnO NPLs' formation featuring the unique bimodal X-type/L-type ligand coordination shell which was derived from an Et_2_Zn/benzamine precursor system.^38^ The above-mentioned systems are rather incompatible with our self-supporting [EtZn(X)]-type (X = monoanionic organic ligand) organometallic precursor system and so far, there is no clear picture of the nucleation and growth of ZnO NCs in this procedure. The standard OSSOM procedure involves initially two competing reactions, oxygenation^39,40^ and hydrolysis^41^ and elucidating the growth mechanism in the system with two competing sources of oxygen, *i.e.*, O_2_ and H_2_O from air, is particularly a challenging and demanding task. Although some well-structurally defined zinc oxo and hydroxide clusters were isolated from molecular scale transformations of organozinc compounds,^42–44^ these aggregates provide limited insight into the factors controlling the growth process.

**Fig. 1:**
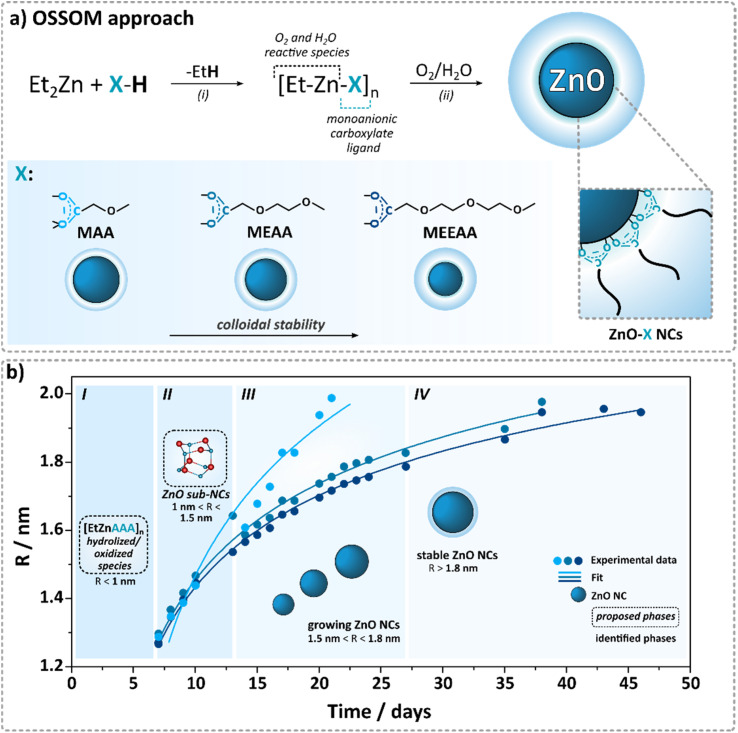
(a) Fabrication of ligand-coated ZnO NCs *via* the OSSOM approach; (b) the growth dynamics of the ZnO NCs over time.

To date, we have conducted extensive studies on a library of carboxylic acid proligands, such as standard mono-^20,21^ and bifunctional carboxylic acids,^22^ including liquid-crystalline-type proligands^24^ and alkoxyacetic acids^20,21^ as miniPEG prototypes. In general, alkylzinc carboxylates,^45–47^ due to their high solubility and stability in most aprotic and dry organic solvents, align well with the requirements of the OSSOM protocol, thereby enhancing the overall efficiency and effectiveness of the ZnO NC fabrication process, yielding mean particle sizes ranging from 2.8 nm to 5.2 nm. For example, these investigations demonstrated that carboxylate-coated NCs exhibit minimal cytotoxicity at concentrations up to 10 μg ml^−1^, and strikingly higher concentrations of NCs with shorter or longer ether tails significantly reduce cell viability.^20,21^ Moreover, the tail length not only influences cytotoxicity but also plays a crucial role in determining the core size of NCs, with shorter ligands typically promoting the formation of larger cores, whereas longer ligands tend to stabilize smaller core structures.^21^ Despite extensive research on the OSSOM procedure, the nucleation and growth of monodisperse ZnO NCs remains not fully understood. Here, to investigate how the growth dynamics of ZnO NCs depend on the length of the carboxylate tail, we monitor the average diameter of the growing NCs over time at ambient temperature, starting from a series of [EtZn(X)]-type carboxylate precursors, specifically incorporating methoxyacetate (MAA), 2-(2-methoxyethoxy)acetate (MEAA), and 2-[2-(2-methoxyethoxy)ethoxy]acetate (MEEAA) ligands. These precursors serve as model self-supporting systems within the OSSOM synthetic procedure (Fig. 1a). In addition, we applied sedimentation velocity analytical ultracentrifugation (SV-AUC) measurements as a stability-indicating method for the characterization of the resulting carboxylate-coated ZnO NCs.

## Results and discussion

### The continuous growth of ZnO NCs

**Fig. 2:**
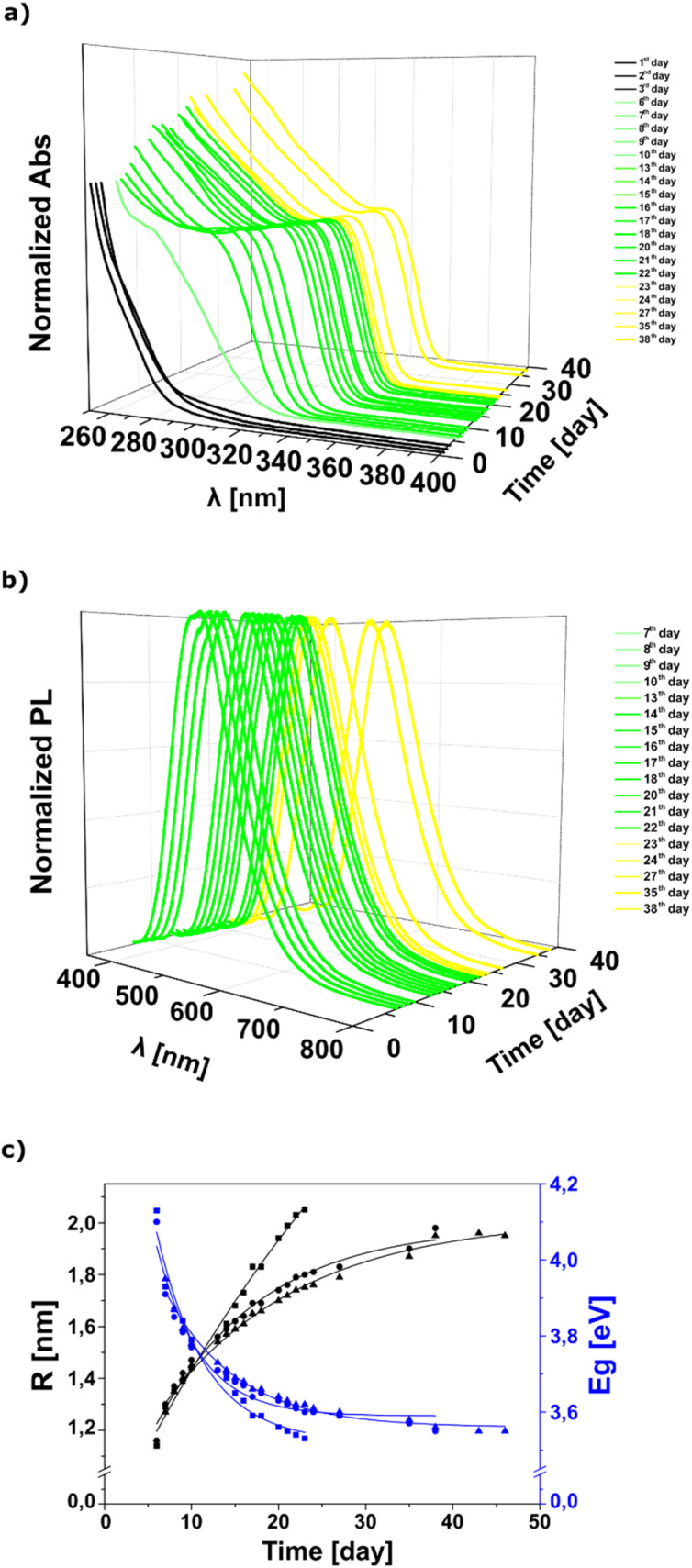
ZnO-MEAA NCs, (a) absorption and (b) fluorescence spectra collected during *in situ* growth of particles in solution as a function of wavelength and time. The difference in line density is due to a non-uniform time between the represented measurements (for data for all ZnO NCs see the ESI†); (c) band gaps from absorption measurements *versus* the particle radius from the Brus formula; in graph (c) data for all the systems studied in this work are presented.

To gain a deeper understanding of the growth of ZnO NCs prepared using the OSSOM synthetic procedure in relation to the ligand structure, we collected electronic absorption and photoluminescence (PL) emission spectra over the growth period. In general, the organometallic self-supporting [EtZn(X)]-type precursors were transformed into ZnO NCs at ambient temperature (approximately 22–26 °C) under significantly restricted air access and at a fixed precursor concentration. The slower aeration, in contrast to the standard OSSOM procedure,^21^ notably extended the transformation time. Using optical spectroscopies, we could follow the discrete changes in optical spectra during the transformation of organometallic precursors into colloidal NCs and distinguish between an early nucleation phase and a late growth phase as well as the subsequent final stabilization in the solution. The absorbance and the PL spectra were collected simultaneously for the same reaction solution for each ZnO NC sample (see Fig. 2 showing the data recorded for ZnO-MEAA NCs and the ESI† for the remaining ZnO-MAA and ZnO-MEEAA NCs). According to the model introduced by Brus *et al.*,^48^ the band gap in semiconductor NCs depends on the NC radius *R* and this relationship can be mathematically described. The *E*_g_ and *R* values calculated using the Brus equation (for details, see the Experimental section) for different ZnO NCs are presented over time in Fig. 2c (and S2–S4†). This relationship exhibits a logarithmic shape and was fitted using the equation *y* = *a* ln(−*b* ln(*x*)) and according to the Bradley model. The adjusted *R*^2^ values indicate high fitting accuracy: 0.88 for ZnO-MAA and 0.99 for ZnO-MEAA and ZnO-MEEAA NCs, respectively (for more details, see the ESI†). Depending on the carboxylate tail length, the slopes of the fitted curves vary along the *X*-axis. The growth curves differ in nanostructure formation dynamics, as evident from comparing the *a* parameter values. The most intensive growth occurs in ZnO NCs coated with the shortest carboxylate ligand (*a* = 1.59), whereas the slowest growth is observed for NCs coated with carboxylates bearing the longest tail (*a* = 1.02). This suggests that nanostructure growth is regulated by the limited availability of oxygen sources, as long ligands shield zinc centers, reducing oxygen access. Additionally, the *b* parameter determines the shape and slope of the curve and can be interpreted as the rate at which the growth slows down. This highlights its crucial role in defining ZnO NC growth kinetics and stabilization. Notably, the ZnO NCs coated with carboxylates bearing the shortest tail exhibit the fastest formation rate, likely due to the initially favorable growth environment. However, over time, the system loses its homogeneity, leading to turbidity in the reaction mixture after 20 days, which significantly hindered further investigations. In contrast, ZnO-MEEAA exhibits a completely different growth dynamic: its initial growth phase is slower, but the lowest *b* coefficient (−1.78) suggests the strong final stabilization of the system.

Undoubtedly, the formation of NCs is governed by two distinct factors: (i) the chemistry of organozinc precursors in the early stage and (ii) interactions between growing nanoparticles and organic ligands in the subsequent stabilization phase. However, the detailed nucleation and growth mechanism of monodisperse ZnO NCs in the OSSOM process remains unclear. Based on our previous molecular-level investigations of interactions of alkylzinc carboxylates and other organozincs with dioxygen^40,49^ and water^41,44^ it is reasonable to assume that the aeration of the *in situ* generated [EtZn(X)]-type precursor^46^ leads to the formation of different intermediate clusters incorporating oxide, oxo/alkoxide and oxo/hydroxide zinc species along with higher organised clusters incorporating preorganised (ZnO)_*x*_ cores (where *x* ≥ 1),^50,51^ including an alkylzinc cluster containing a [Zn_10_O_4_]^12+^ supertetrahedron core^51^ among others^43^ and stabilized using organic ligands (see the area designated as I in Fig. 1b). The latter complexes may act as nucleation sites that facilitate further growth, serving as templates for subsequent wurtzite-type core nanoparticle formation. At this stage, ZnO NCs grow through the controlled addition of individual oxo-based building blocks. Thus, after the first 6–7 days, ZnO sub-NCs with characteristic surface-trap emission and *D* = 2*R* = 2.28, 2.32, 2.54 nm (*E*_g_ = 4.13, 4.10, 3.95 eV) were found for ZnO-MAA, ZnO-MEAA, and ZnO-MEEAA NCs, respectively (see the area designated as II on Fig. 1b). Then, the UV-Vis and PL spectra shifted towards longer wavelengths and the ZnO NC size was steadily increasing up to *D* = 4.10, 3.96, 3.60 nm (*E*_g_ = 3.53, 3.55, 3.55 eV; see the areas designated as III and IV in Fig. 1b). Subsequently, a plateau is observed on the graph illustrating the magnitude over time (see the area designated as IV in Fig. 1b), which can be interpreted as a stage of NC stabilization in solution, *i.e.* producing the thermodynamically lowest energy crystal structure determined by the character of the organic–inorganic interface. Thus, we decided to terminate our study after 23, 38, and 46 days for ZnO-MAA, ZnO-MEAA and ZnO-MEEAA NCs, respectively (note that the calculated data at this NCs size stage correlate well with data previously reported for these systems^21^). To further verify reproducibility, we conducted PXRD and STEM analyses (for details, see the Experimental section and ESI†), confirming consistency with previously studied NCs and demonstrating the high reproducibility of the OSSOM method (see Fig. S6 and S7, ESI†). Additionally, ZnO-MEAA NCs were selected as a model system to assess stability in various organic solvents and water (see Table S3 and Fig. S8 in the ESI†). These tests demonstrated outstanding dispersibility of ZnO NCs across a broad range of media.

Overall, our study substantiates the “living growth” nature of ZnO NC formation, emphasizing the key role of thermodynamics in stabilizing the NC structure and determining the preferred crystalline forms in a given environment. It is justifiable to assert that ligand interactions, along with the characteristics of the organic–inorganic interface, significantly influence how NCs attain a thermodynamic equilibrium state. This process ultimately leads to the formation of stable monodispersed structures with the lowest energy configurations.

### Study of solution properties of colloidal ZnO NCs using the AUC method

Analytical centrifugation (AUC) is one of the oldest analytical methods used to analyze biological macromolecules such as proteins and nucleic acids and their complexes.^52^ Over the last decade, AUC has proven highly useful for characterizing synthetic materials, including polymers, colloids, and nanoparticles.^53–55^ This method is successfully used, among others, to determine the properties of these molecules. Due to the growing importance of nanoparticles in various applications, the standardization of these particles and the assessment of their stability are crucial. A relatively large number of studies describe simpler systems that are mixtures of monodisperse standard nanoclusters. More serious interpretation problems begin in the case of polydisperse systems composed of core molecules and their attached side chains. In such cases, calculating even the basic parameters needed for advanced analysis, such as partial specific volume, is very difficult.^56,57^

**Fig. 3:**
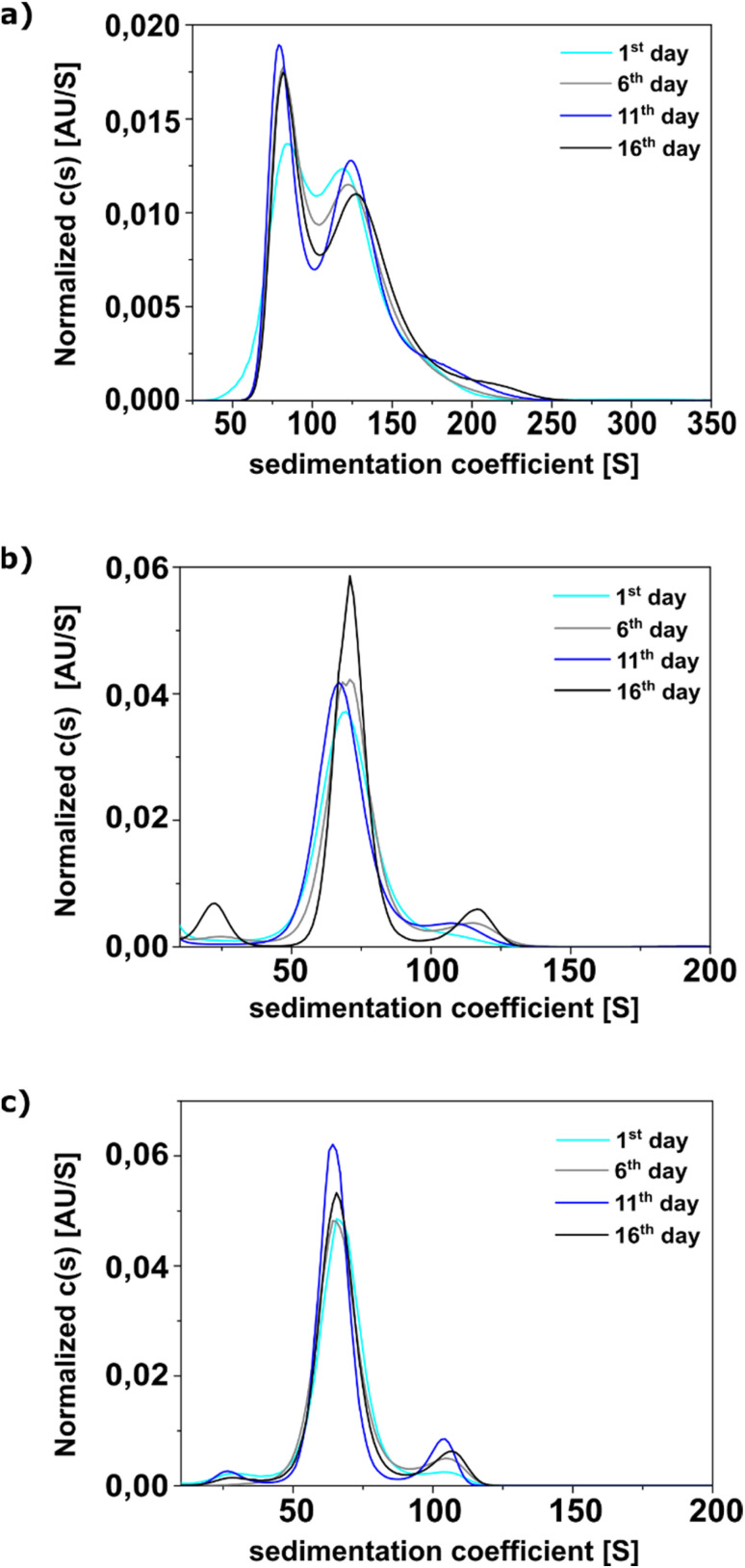
Sedimentation coefficient distribution *c*(*s*) from the analysis of the ZnO NCs with the different lengths of the side chains attached to the ZnO core, *i.e.*, ZnO-MAA (a), ZnO-MEAA (b) and ZnO-MEEAA NCs (c), respectively. The experiment was carried out in 5-day intervals. Signal intensity fluctuations in individual experiments resulted from the need to store the rotor in a refrigerator (4 °C) and install the centrifuge optical system each time before the experiment. For clarity, the data have been normalized to the area (under the *c*(*s*) distribution).

According to our previous work, the average hydrodynamic diameters of ZnO NCs in THF were approximately 11–12 nm, and shelf-life studies confirmed a high colloidal stability of these systems.^21^ To further investigate this phenomenon, we employed AUC to assess how ligand-coated ZnO NCs behave in THF under centrifugal forces. This approach allows us to evaluate their stability and aggregation tendencies over time by comparing the sedimentation profiles of each sample. For this purpose, each of them was scanned four times at intervals of five days. Between experiments, the cells were kept in a rotor at 4 °C. The results illustrating changes in the profile over time are shown in Fig. 3 (and S9–S11 in the ESI†).

To standardize the calculations, the percentage contribution of each peak relative to the total *c*(*s*) signal in a given experiment was analysed (see Table S3 in the ESI†). Based on the peak positions, three sedimentation coefficient ranges were determined for each sample: 5–60*S*, 60–180*S*, and 180–260*S* for ZnO-MAA; 5–40*S*, 40–100*S*, and 100–140*S* for ZnO-MEAA and 5–40*S*, 40–90*S*, and 90–120*S* for ZnO-MEEAA NCs. In the case of ZnO-MAA, the middle range (60–180*S*) contained two closely spaced peaks, making it possible to estimate only their combined contribution (Fig. 3a); therefore, they were analyzed together.

In each case, the simplest profile was observed in the first spin. Moreover, a clear dominance of populations within the central *S* range was observed. In the first experiment, their proportion reached 95.5% (ZnO-MAA), 92.7% (ZnO-MEAA), and 92.4% (ZnO-MEEAA). During subsequent centrifugations, a general decrease in this range was noted, ranging from a few percent for ZnO-MAA to several percent for ZnO-MEAA NCs. During subsequent centrifugations, an increase was observed in the division of the sedimenting fraction that was the fastest and located in the third range. This was especially true for ZnO-MEAA and ZnO-MEEAA samples for which it reached a value of about 10%. These samples were also characterized by different dynamics of growth of this fraction. A significant increase was observed already during the second centrifugation, and in subsequent centrifugations the level remained relatively constant. In the case of the ZnO-MAA sample, the growth of the division occurred gradually reaching a maximum in the last centrifugation. It is also noteworthy that in the case of the central range of the ZnO-MAA sample, the reciprocal of each peak was stable throughout the experiment, amounting to 39.2, 40.3, 40.9, and 39.0% for the first peak (max. 84*S*) and 56.6, 56.7, 54.2, and 55.2% for the second peak (max. 129*S*), respectively. Despite 16 days of the experiment and 4 independent centrifugations, relatively little reorganization of the sedimentation profile of nanoparticles was observed. The basic change is a slight increase in the share of the population with the highest sedimentation coefficients. There was still a clear dominance of the main population. This proves the high stability of NCs obtained by the OSSOM method. There was also no rapid loss of signal intensity, which could indicate progressive core–core type aggregation or degradation of the nanostructures. It is interesting that the increase in the third fraction is proportional to the length of the side chains of nanoparticles and is the largest in the case of ZnO-MEEAA.

It is reasonable that starting with the first centrifugation, when the samples are the freshest, *i.e.*, right after the final growth stage, they exhibit the highest homogeneity. Over time, however, we can observe the formation of structures that lead to separation into distinct fractions. For instance, two or three nanocrystals may interact, resulting in a greater mass that causes them to sediment faster. Our data indicate that the chance of molecules interacting is proportional to the length of the side chains. On the other hand several nanocrystals interacting with each other and forming a soft-type aggregate create additional voids between them, causing slower sedimentation due to the increased surface area of the system. In the case of the OSSOM-derived ZnO NCs, this phenomenon occurs to a relatively minimal extent and is practically stopped in a short time by creating a thermodynamic equilibrium. It also worth noting that analytical centrifugation is a precise method that is much more sensitive to detecting such changes compared to other standard analytical techniques.

## Experimental section

### General remarks

All manipulations involving air- and moisture-sensitive diethylzinc (Et_2_Zn) were conducted under a nitrogen atmosphere using standard Schlenk techniques. THF was purified and dried using a MBraun Solvent Purification System (SPS) and stored over 3 Å molecular sieves. All reagents were used as received; these include methoxyacetic acid (Sigma Aldrich, 98%), 2-(2-methoxyethoxy)acetic acid (Sigma Aldrich, technical grade), 2-[2-(2-methoxyethoxy)-ethoxy)acetic acid (Sigma Aldrich, technical grade), and Et_2_Zn (ABCR, 95% solution in hexane; note that for further research 2.1 M solution of Et_2_Zn in hexane was prepared).

### General procedure for ZnO NC preparation

ZnO NCs were prepared according to the previously described OSSOM procedure.^20,21^ Briefly, 2 mmol of Et_2_Zn (0.95 mL of a 2.1 M solution in hexane) was added to a stirred solution of the selected organic pro-ligand (2 mmol) in THF (10 ml) at −78 °C. Then, the reaction mixture was warmed up to room temperature and stirred vigorously for an additional 6 h. The preparation of ZnO NCs was realized through the exposure of a solution of the *in situ* prepared [EtZn(X)]-type precursor (where X = selected monoanionic alkoxyacetate ligand, *i.e.*, MAA, MEAA, and MEEAA, respectively) in THF to air at ambient temperature. Stable and redispersible ZnO NCs were obtained within 7 days. The optical characteristics of the as-received material and the corresponding calculated core sizes are consistent with those of previously reported alkoxyacetate-coated ZnO NCs,^20,21^*i.e.*, ZnO-MAA NCs (*λ*_abs_ = 336 nm; *λ*_em_ = 523 nm; FWHM = 128,3; *d* = 4,08 ± 0,49 nm; *E*_g_ = 3.54 eV); ZnO-MEAA NCs (*λ*_abs_ = 331 nm; *λ*_em_ = 514 nm; FWHM = 125.1; *d* = 3.74 ± 0.53 nm; *E*_g_ = 3.58 eV); ZnO-MEEAA NCs (*λ*_abs_ = 328 nm; *λ*_em_ = 510 nm; FWHM = 123.2; *d* = 3.55 ± 0.56 nm; *E*_g_ = 3.61 eV). Moreover, the obtained sizes show excellent agreement with the values calculated from data acquired using other analytical methods (*i.e.*, PXRD and STEM, *vide infra*).

### Characterization methods

Size and shape of the ZnO NCs were examined using a Cs-corrected Scanning Transmission Electron Microscope (HITACHI HD2700, 200 kV). For STEM measurements ZnO NC samples were drop-cast (THF or DMSO solution) onto 300-mesh, holey carbon-coated copper grids (Quantifoil). The STEM micrographs show well-dispersed and spherically shaped ZnO-MAA, ZnO-MEAA and ZnO-MEEAA NCs with a mean core diameter of 5.0 ± 0.9, 4.5 ± 0.8 and 4.2 ± 0.7 nm, respectively.

Powder XRD data were collected on an Empyrean diffractometer (PANalytical) by employing Ni-filtered CuKα radiation from a copper sealed tube charged with 40 kV voltage and 40 mA current in Bragg–Brentano geometry with beam divergence of 1 deg. in the scattering plane. Diffraction patterns were measured in the range of 20–80 degrees of scattering angle by step scanning with a step of 0.008°. The average core sizes calculated from the Scherrer equation *d*_c_ = (*kλ*)/*β* cos *θ*, where *d*_c_ – diameter of the inorganic core; *k* – Scherrer's constant (crystallite-shape factor), *k* = 0.89; *λ* – wavelength of the X-rays, *λ* = 1.54 Å; *θ* – Bragg diffraction angle; *β* – full-width at half-maximum of the X-ray diffraction peak, which is 3.00 ± 0.24 nm, 3.23 ± 0.39 nm and 3.23 ± 0.40 nm for ZnO-MAA, ZnO-MEAA and ZnO-MEEAA NCs, respectively.

### UV-vis/PL investigation of growth of ligand-coated ZnO NCs

The formation and growth of ZnO NCs were investigated using UV-Vis and PL spectroscopy simultaneously. Optical absorption (UV-vis) spectra were collected on a Hitachi U-2910 spectrophotometer. A standard quartz cell (Hellma) with a 10 mm path length was used and rinsed with proper solvent before each run. Photoluminescence (PL) measurements were carried out using a HITACHI fluorescence spectrophotometer F-7000.

In a standard experiment, the generated *in situ* organozinc [EtZn(X)]-type precursor (X = MAA, MEAA, MEEAA, respectively) in THF was subjected to controlled and limited exposure to air conditions. Then, the optical properties of the reaction mixture were monitored by UV-vis and PL spectroscopy. The measurements of absorption and photoluminescence were performed day by day over an extended period (up to 23, 38 and 46 days for ZnO-MAA, ZnO-MEAA and ZnO-MEEAA NCs, respectively). The limited access of atmospheric air to the reaction solution was ensured when only the tap in the Schlenk vessel was open. Each time, 1 ml of pure solvent was poured into the standard quartz cell and then the reaction mixture was added dropwise until the absorbance reached the level of 1 at a wavelength of 250 nm. For such a concentration of the sample, the maximum absorbance was determined and the emission spectra were recorded. All source data were normalized according to a single scheme. The maxima of the absorption and photoluminescence spectra were identified. These values were normalized to 1 by dividing each maximum by itself. Subsequently, each data point in the table of original values was divided by the determined maximum value. New graphs were created from the resulting normalized data, which are presented in this article. Subsequently, the radii (*R*) and particle sizes (*D* = 2*R*) for all as-prepared ZnO samples were calculated from experimentally determined optical parameters using the Brus formula,^58,59^
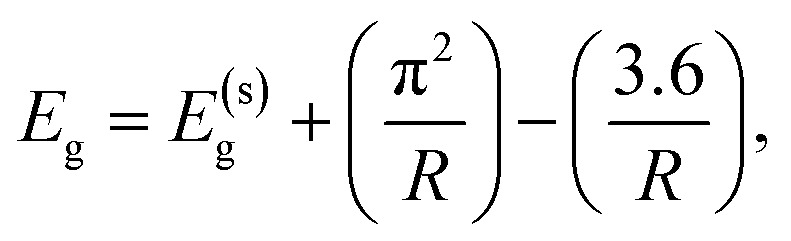
 where *E*_g_ – cluster band gap energy calculated from absorption spectrum using the Tauc relation,^60,61^*E*^(s)^_g_ – solid-state band gap energy equal to 3.44 eV,^62^ and *R* – NC radius. Our results are presented in Table S1 and Fig. S2–S4.† The experimental data for ZnO NCs were fitted using a logarithmic curve (Bradley model) provided in OriginLab software.

### Analytical centrifugation

To acquire experimental data about sedimentation coefficient distribution and solution stability of the ZnO NCs, sedimentation velocity (SV) measurements were carried out in a Beckman–Coulter ProteomeLab XL-I analytical ultracentrifuge (Indianapolis, USA) equipped with an An-60, 4-hole analytical rotor and 12 mm path length, double-sector solid aluminum centerpieces (with extra gaskets on both sides of the cells). ZnO NCs were measured in THF. Cells containing 400 μl of sample and 410 μl of solvent were centrifuged at 10 000 rpm and monitored by UV absorbance at 347 nm at 20 °C, using continuous scan mode and a radial spacing of 0.003 cm. Four centrifugations of each sample were performed at 5-day intervals. In the period between the experiments, the rotor was stored at 4 °C. The sample cells were not removed from the rotor, but the rotor was shaken vigorously for 5 minutes before and after each centrifugation. Data were analyzed using the “continuous *c*(*s*) distribution” model of the SEDFIT program,^63^ with confidence level (*F*-ratio) specified to 0.68. Partial specific volume of the ZnO core was assumed to be 0.1783 cm^3^ g^−1^. The results were plotted using the GUSSI graphical program.^64^ To ensure a better comparison of the results of individual centrifugations, they were normalized (in GUSSI) in relation to the total absorption of the samples (*i.e.* the area under the *c*(*s*) distribution).

## Conclusions

Understanding the growth and stabilization mechanisms of metal oxide nanostructures is essential for the rational-by-design of synthetic procedures toward nanostructures of desired physicochemical characteristics. Recently, we have developed the facile OSSOM method employing [EtZn(X)]-type precursors, which affords reproducibly unprecedented high-quality colloidal quantum-sized ZnO crystals. The resulting NCs are coated with strongly anchored X-type organic ligands and their character strongly determines the NC size and functionality. Nevertheless, the controlled growth dynamics in the OSSOM process have not yet been thoroughly investigated. In our studies involving a series of [EtZn(X)]-type precursors with carboxylate ligands of varying tail lengths, we demonstrated that the growth of ZnO NCs in this self-supporting organometallic process follows a “living growth” mechanism. We observed that as the ligand length increases, the growth rate of NCs decreases due to more limited oxygen access, while system stabilization improves, resulting in stable colloidal solutions.

Although this aspect was not studied conclusively, our results suggest that the formation of NCs proceeds along several consecutive steps which include a complex sequence of precursor reactions, nucleation, and growth processes. The observed rapid initial nucleation, followed by growth and gradual stabilization of the final ZnO NCs, leads to the formation of the thermodynamically lowest-energy crystal structure. Furthermore, our data on the growth dynamics of NCs support the conclusion that we achieve specific NC sizes, which are thermodynamically controlled by the characteristics of the organic–inorganic interface and the character of the applied organic ligand. Detailed analysis using analytical ultracentrifugation reveals that the final colloidal solution consists predominantly of single fractions. Although our experiments are based on very simple kinetic and thermodynamic models, new insights into fundamental and general principles of NC formation using the OSSOM procedure can be gained, including the role of the length of the carboxylate tail in the growth dynamics of ZnO NCs. Thus, these findings should facilitate further design of colloidal nanostructures.

## Data availability

The data supporting this article have been included as part of the ESI.†

## Conflicts of interest

There are no conflicts to declare.

## Supplementary Material

NA-OLF-D4NA00933A-s001

## References

[cit1] Djurišić A. B., Chen X., Leunga Y. H., Man Ching Ng A. (2012). J. Mater. Chem..

[cit2] Raha S., Ahmaruzzaman Md. (2022). Nanoscale Adv..

[cit3] Chavan R. D., Wolska-Pietkiewicz M., Prochowicz D., Jędrzejewska M., Tavakoli M. M., Yadav P., Hong C. K., Lewiński J. (2022). Adv. Funct. Mater..

[cit4] van Embden J., Gross S., Kittilstved K. R., Della Gaspera E. (2023). Chem. Rev..

[cit5] Wang Z., Bockstaller M. R., Matyjaszewski K. (2021). ACS Mater. Lett..

[cit6] Spanhel L., Anderson M. A. (1991). J. Am. Chem. Soc..

[cit7] Spanhel L. (2006). J. Sol-Gel Sci. Technol..

[cit8] Meulenkamp E. A. (1998). J. Phys. Chem. B.

[cit9] Viswanatha R., Amenitsch H., Sarma D. D. (2007). J. Am. Chem. Soc..

[cit10] Pacholski C., Kornowski A., Weller H. (2002). Angew. Chem., Int. Ed..

[cit11] Caetano B. L., Briois V., Pulcinelli S. H., Meneau F., Santilli C. V. (2016). J. Phys. Chem. C.

[cit12] Herbst M., Hofmann E., Förster S. (2019). Langmuir.

[cit13] Olejnik-Fehér N., Jędrzejewska M., Wolska-Pietkiewicz M., Lee D., De Paëpe G., Lewiński J. (2024). Small.

[cit14] Lee D., Wolska-Pietkiewicz M., Badoni S., Grala A., Lewiński J., De Paëpe G. (2019). Angew. Chem., Int. Ed..

[cit15] Monge M., Kahn M. L., Maisonnat A., Chaudret B. (2003). Angew. Chem., Int. Ed..

[cit16] Kahn M. L., Monge M., Collière V., Senocq F., Maisonnat A., Chaudret B. (2005). Adv. Funct. Mater..

[cit17] Kahn M. L., Cardinal T., Bousquet B., Monge M., Jubera V., Chaudret B. (2006). ChemPhysChem.

[cit18] Wolska-Pietkiewicz M., Jędrzejewska M., Tokarska K., Wielgórska J., Chudy M., Grzonka J., Lewiński J. (2023). Chem. Eng. J..

[cit19] Jędrzejewska M., Wolska-Pietkiewicz M., Drużyński Z., Lewiński J. (2023). J. Mater. Chem. C.

[cit20] Wolska-Pietkiewicz M., Tokarska K., Grala A., Wojewódzka A., Chwojnowska E., Grzonka J., Cywiński P. J., Kruczała K., Sojka Z., Chudy M., Lewiński J. (2018). Chem.–Eur. J..

[cit21] Wolska-Pietkiewicz M., Tokarska K., Wojewódzka A., Wójcik K., Chwojnowska E., Grzonka J., Cywiński P. J., Chudy M., Lewiński J. (2019). Sci. Rep..

[cit22] Grala A., Wolska-Pietkiewicz M., Wróbel Z., Ratajczyk T., Kuncewicz J., Lewiński J. (2018). Mater. Chem. Front..

[cit23] Grala A., Wolska-Pietkiewicz M., Danowski W., Wróbel Z., Grzonka J., Lewiński J. (2016). Chem. Commun..

[cit24] Paczesny J., Wolska-Pietkiewicz M., Binkiewicz I., Wróbel Z., Wadowska M., Matuła K., Dzięcielewski I., Pociecha D., Smalc-Koziorowska J., Lewiński J., Hołyst R. (2015). Chem.–Eur. J..

[cit25] Wolska-Pietkiewicz M., Grala A., Justyniak I., Hryciuk D., Jędrzejewska M., Grzonka J., Kurzydłowski K., Lewiński J. (2017). Chem.–Eur. J..

[cit26] Chwojnowska E., Wolska-Pietkiewicz M., Grzonka J., Lewiński J. (2017). Nanoscale.

[cit27] Terlecki M., Badoni S., Leszczyński M. K., Gierlotka S., Justyniak I., Okuno H., Wolska-Pietkiewicz M., Lee D., De Paëpe G., Lewiński J. (2021). Adv. Funct. Mater..

[cit28] Chwojnowska E., Kowalska A. A., Kamińska A., Lewiński J. (2024). ACS Appl. Mater. Interfaces.

[cit29] Cieślak A. M., Pavliuk M. V., D'Amario L., Abdellah M., Sokołowski K., Rybinska U., Fernandes D. L. A., Leszczyński M. K., Mamedov F., El-Zhory A. M., Föhlinger J., Budinská A., Wolska-Pietkiewicz M., Hammarström L., Lewiński J., Sá J. (2016). Nano Energy.

[cit30] Thanh N. T. K., Maclean N., Mahiddine S. (2014). Chem. Rev..

[cit31] Xia Y., Xia X., Peng H.-C. (2015). J. Am. Chem. Soc..

[cit32] Ji S., Abbas H. G., Kim S. Y., Lee H. C., Lee K., Li S., Choe S., Ahn H., Ringe S., Yang J. (2025). Small Sci..

[cit33] Wood A., Giersig M., Hilgendorff M., Vilas-Campos A., Liz-Marzan L. M., Mulvaney P. (2003). Aust. J. Chem..

[cit34] Bilecka I., Elser P., Niederberger M. (2009). ACS Nano.

[cit35] Liang M.-K., Limo M. J., Sola-Rabada A., Roe M. J., Perry C. C. (2014). Chem. Mater..

[cit36] Wang Y., Coppel Y., Lepetit C., Marty J.-D., Mingotaud C., Kahn M. L. (2021). Nanoscale Adv..

[cit37] Zhao Z., Wang Y., Delmas C., Mingotaud C., Marty J.-D., Kahn M. L. (2021). Nanoscale Adv..

[cit38] Badoni S., Terlecki M., Carret S., Poisson J. F., Charpentier T., Okuno H., Wolska-Pietkiewicz M., Lee D., Lewiński J., De Paëpe G. (2024). J. Am. Chem. Soc..

[cit39] Lewiński J., Marciniak W., Lipkowski J., Justyniak I. (2003). J. Am. Chem. Soc..

[cit40] Kubisiak M., Zelga K., Bury W., Justyniak I., Budny-Godlewski K., Ochal Z., Lewiński J. (2015). Chem. Sci..

[cit41] Bury W., Krajewska E., Dutkiewicz M., Sokołowski K., Justyniak I., Kaszkur Z., Kurzydłowski K. J., Płocinski T., Lewinski J. (2011). Chem. Commun..

[cit42] Prochowicz D., Sokołowski K., Lewiński J. (2014). Coord. Chem. Rev..

[cit43] Terlecki M. , Wolska-Pietkiewicz M., Lewiński J., in Nanomaterials via Single-Source Precursors, Chapter 8 - Organometallic single-source precursors to zinc oxide-based nanomaterials, ed. A. W. Apblett, A. R. Barron and A. F. Hepp, Elsevier, 2022, pp. 245–279

[cit44] Pike S. D., White E. R., Shaffer M. S. P., Williams C. K. (2016). Nat. Commun..

[cit45] Grala A., Wolska-Pietkiewicz M., Wojewódzka A., Dabergut M., Justyniak I., Lewiński J. (2015). Organometallics.

[cit46] Orchard K. L., Harris J. E., White A. J. P., Shaffer M. S. P., Williams C. K. (2011). Organometallics.

[cit47] Said S. A., Roberts C. S., Kyung Lee J., Shaffer M. S. P., Williams C. K. (2021). Adv. Funct. Mater..

[cit48] Efros A. L., Brus L. E. (2021). ACS Nano.

[cit49] Lewiński J., Bury W., Dutkiewicz M., Maurin M., Justyniak I., Lipkowski J. (2008). Angew. Chem., Int. Ed..

[cit50] Zelga K., Leszczyński M., Justyniak I., Kornowicz A., Cabaj M., Wheatley A. E. H., Lewiński J. (2012). Dalton Trans..

[cit51] Leszczyński M. K., Justyniak I., Zelga K., Lewiński J. (2017). Dalton Trans..

[cit52] Jibaja Valderrama O., Nischang I. (2021). Anal. Chem..

[cit53] Cölfen H. (2023). Colloid Polym. Sci..

[cit54] Planken K. L., Cölfen H. (2010). Nanoscale.

[cit55] González-Rubio G., Hilbert H., Rosenberg R., Ni B., Fuhrer L., Cölfen H. (2021). Nanomaterials.

[cit56] Walter J., Gorbet G., Akdas T., Segets D., Demeler B., Peukert W. (2017). Analyst.

[cit57] Urban M. J., Holder I. T., Schmid M., Fernandez Espin V., Garcia de la Torre J., Hartig J. S., Cölfen H. (2016). ACS Nano.

[cit58] Brus L. (1986). J. Phys. Chem..

[cit59] Monticone S., Tufeu R., Kanaev A. V. (1998). J. Phys. Chem. B.

[cit60] Tauc J., Menth A. (1972). J. Non-Cryst. Solids.

[cit61] Osuwa J. C., Oriaku C. I., Kalu I. A. (2009). Chalcogenide Lett..

[cit62] Lizandara-Pueyo C., van den Berg M. W. E., De Toni A., Goes T., Polarz S. (2008). J. Am. Chem. Soc..

[cit63] Schuck P. (2000). Biophys. J..

[cit64] Brautigam C. A. (2015). Methods Enzymol..

