# Supplementary material for: A new insight into the fabrication of colloidal isotropic ZnO nanocrystals by an organometallic approach
Source: Nanoscale Adv. 2025 Mar 12;7(9):2677–85. doi: 10.1039/d4na00933a (PMC11938107; doi:10.1039/d4na00933a)
Supplement: NA-007-D4NA00933A-s001 [file NA-007-D4NA00933A-s001.pdf]

Supplementary Information (SI) for Nanoscale Advances.
This journal is © The Royal Society of Chemistry 2025

Supplementary Information

A new insight into the fabrication of uniform ZnO nanocrystals by organometallic approach

Anna Wojewódzka,[a] Małgorzata Wolska-Pietkiewicz,*[a] Roman H. Szczepanowski,[b] Maria Jędrzejewska[a], Karolina Zelga[a] and Janusz Lewiński*[a,c]

a. Faculty of Chemistry, Warsaw University of Technology, Noakowskiego 3, 00-664 Warsaw, Poland

* E-mail: malgorzata.pietkiewicz@pw.edu.pl; janusz.lewinski@pw.edu.pl

b. International Institute of Molecular and Cell Biology, Ks. Trojdena Street 4, 02-109 Warsaw, Poland

c. Institute of Physical Chemistry, Polish Academy of Sciences, Kasprzaka 44/52, 01-224 Warsaw, Poland

1. Steady-state spectroscopy

a)

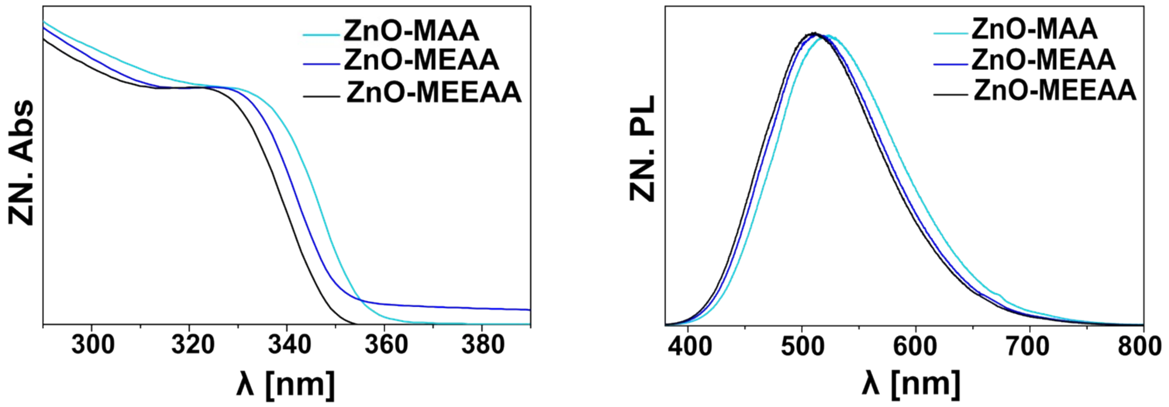

Figure S1. Normalized absorption (a) and emission (b) spectra of as-prepared ZnO-MAA, ZnO-MEAA and ZnO-MEEAA NCs collected in THF.

2. The estimation of the NCs size from the optical spectroscopy

Table S1. Basic physicochemical parameters such as maximum values of absorption and photoluminescence, the diameter of the inorganic ZnO core, the energy gap and half-width of the emission spectrum. The data presented below are consistent with the previously reported data.[1]

Solvent	ZnO NCs	Max Abs. [nm]	Max PL [nm]	Core Size [nm]	Band Gap [eV]	FWHM [nm]
THF	ZnO-MAA	336	523	4.08 ± 0.49	3,54	128.3
	ZnO-MEAA	331	514	3.74 ± 0.53	3,58	125.1
	ZnO-MEEAA	328	510	3.55 ± 0.56	3,61	123.2

[1] M. Wolska-Pietkiewicz, K. Tokarska, A. Wojewódzka, K. Wójcik, E. Chwojnowska, J. Grzonka, P. J. Cywiński, M. Chudy, J. Lewiński, *Scientific Reports*, **2019**, *9*, 18071

3. The kinetics of the growth of ligand-coated ZnO NCs synthesized using organometallics approach

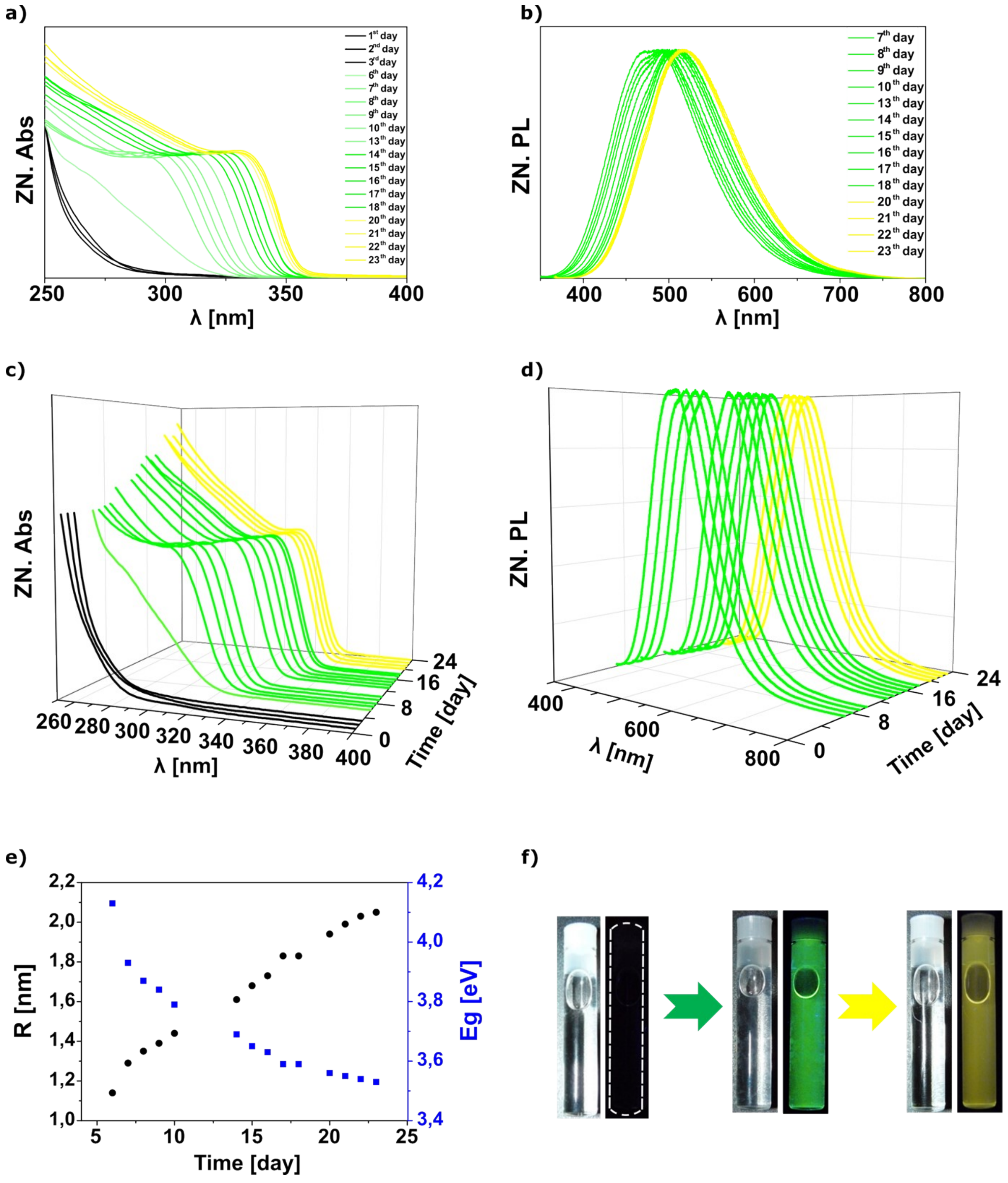

Figure S2. a), b) Two- and c), d) three-dimensional representation of absorption and PL emission spectra as a function of wavelength and time measured on in-situ growing ZnO-MAA in THF solution. The difference in line density is due to a non-uniform time between measurements; e) Band gaps from absorption measurements versus particle radius from Brus formula; f) photos of observed changes in luminescence color of nanocrystals.

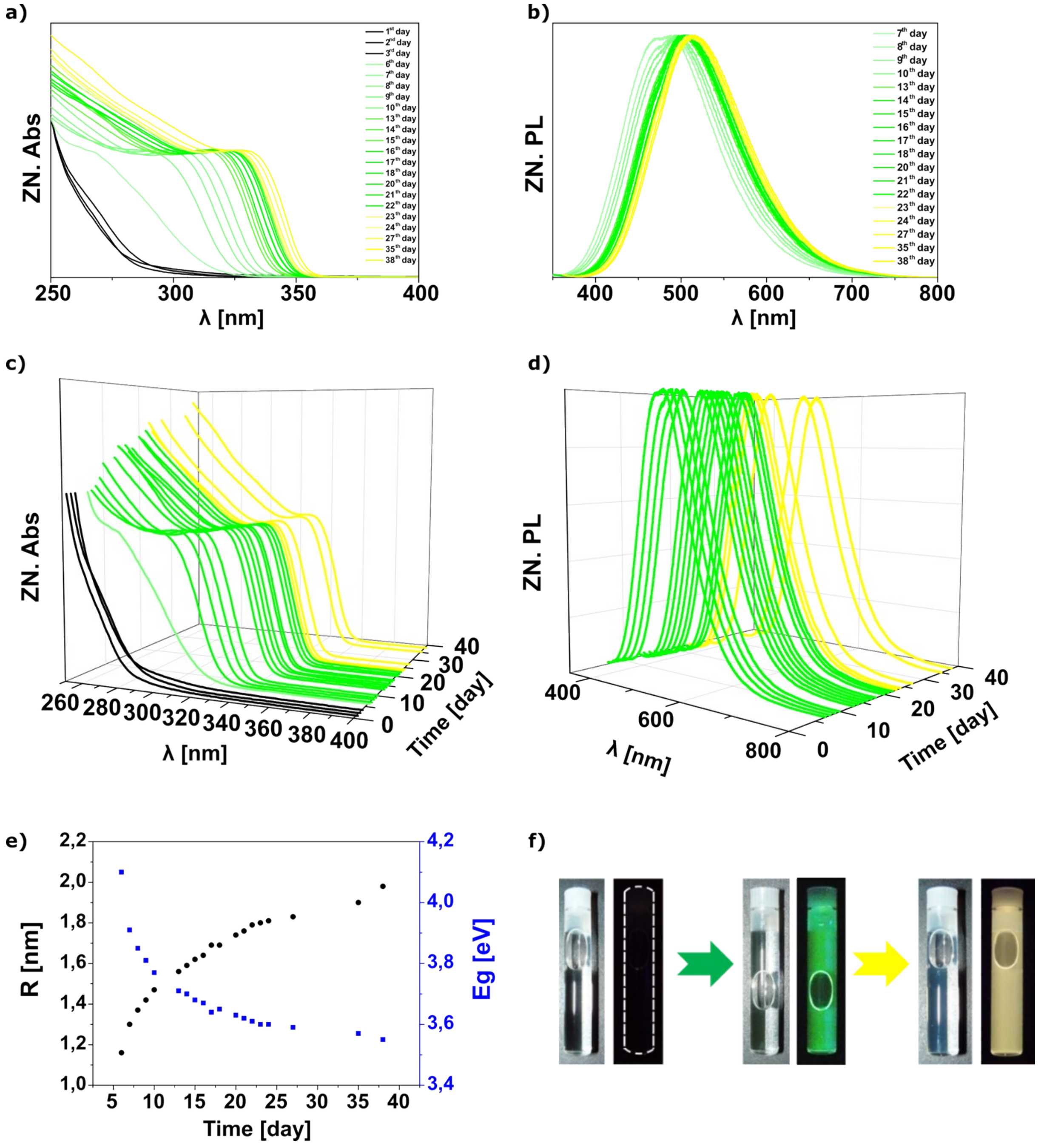

Figure S3. a), b) Two- and c), d) three-dimensional representation of absorption and PL emission spectra as a function of wavelength and time measured on in-situ growing ZnO-MEAA in THF solution. The difference in line density is due to a non-uniform time between measurements; e) Band gaps from absorption measurements versus particle radius from Brus formula; f) photos of observed changes in luminescence color of nanocrystals.

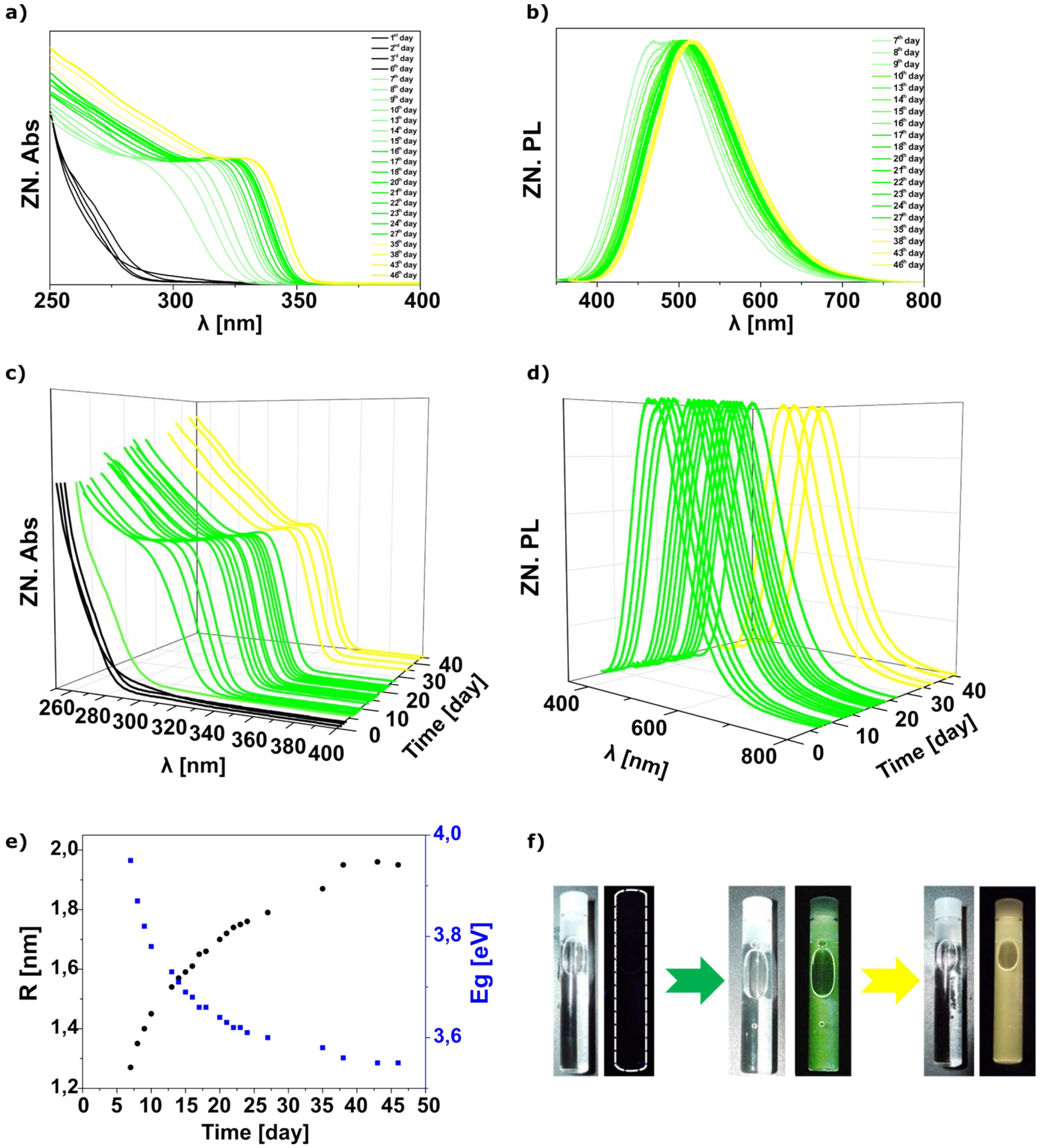

Figure S4. a), b) Two- and c), d) three-dimensional representation of absorption and PL emission spectra as a function of wavelength and time measured on in-situ growing ZnO-MEEAA in THF solution. The difference in line density is due to a non-uniform time between measurements; e) Band gaps from absorption measurements versus particle radius from Brus formula; f) photos of observed changes in luminescence color of nanocrystals.

The experimental data for ZnO NCs was fitted using a logarithmic curve (Bradley model) provided in OriginLab software.

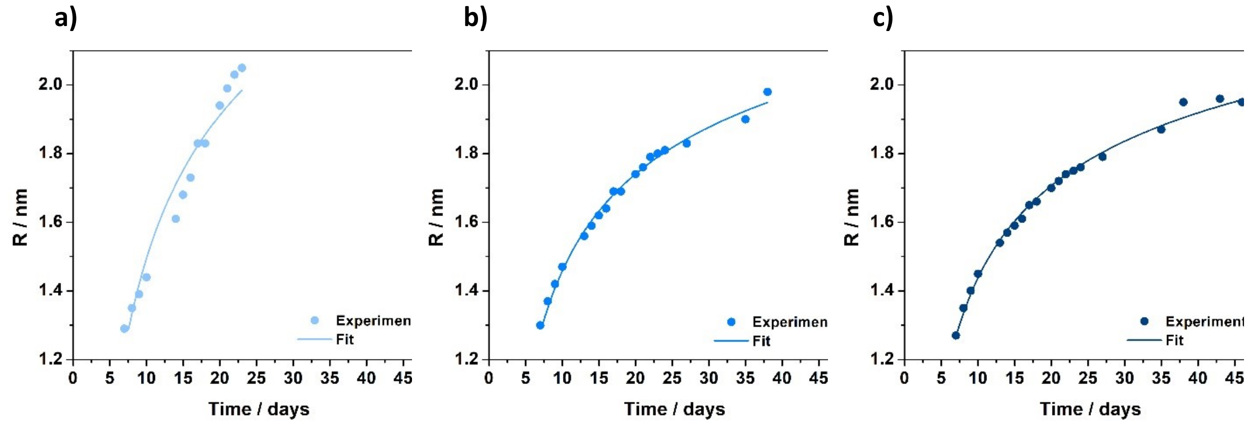

Figure S5. The fitted formation kinetics of a) ZnO-**MAA**, b) ZnO-**MEAA**, and c) ZnO-**MEEAA** NCs.

Table S2. The summary of fitting parameters for formation kinetics of ZnO NCs.

**Sample**	**Fitting model**	**Fitting formula**	R^2
**ZnO-MAA NCs**		**y = *a*ln(-*b*ln(x))**<br>*a* = 1.59 ± 0.09<br>*b* = -1.11 ± 0.07	0.96
**ZnO-MEAA NCs**	Logarithmic (Bradley)	**y = *a*ln(-*b*ln(x))**<br>*a* = 1.07 ± 0.02<br>*b* = -1.692 ± 0.05	0.99
**ZnO-MEEAA NCs**		**y = *a*ln(-*b*ln(x))**<br>a = 1.02 ± 0.02<br>b = -1.78 ± 0.05	0.99

4. PXRD and HRTEM analyses

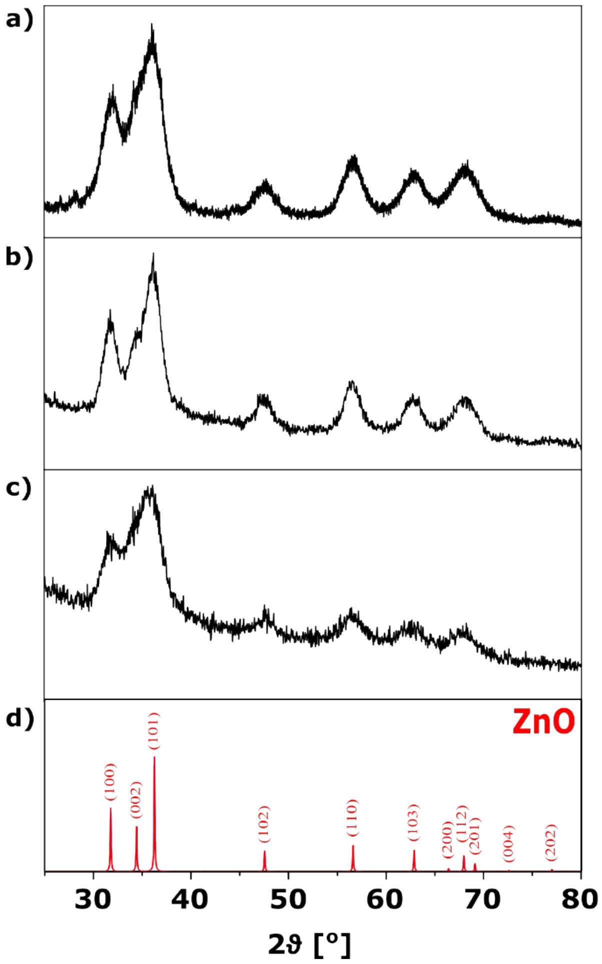

Figure S6. a), b) Powder X-ray diffraction pattern of ZnO-MAA, ZnO-MEAA and ZnO-MEEAA NCs confirming ZnO wurtzite crystalline structure.

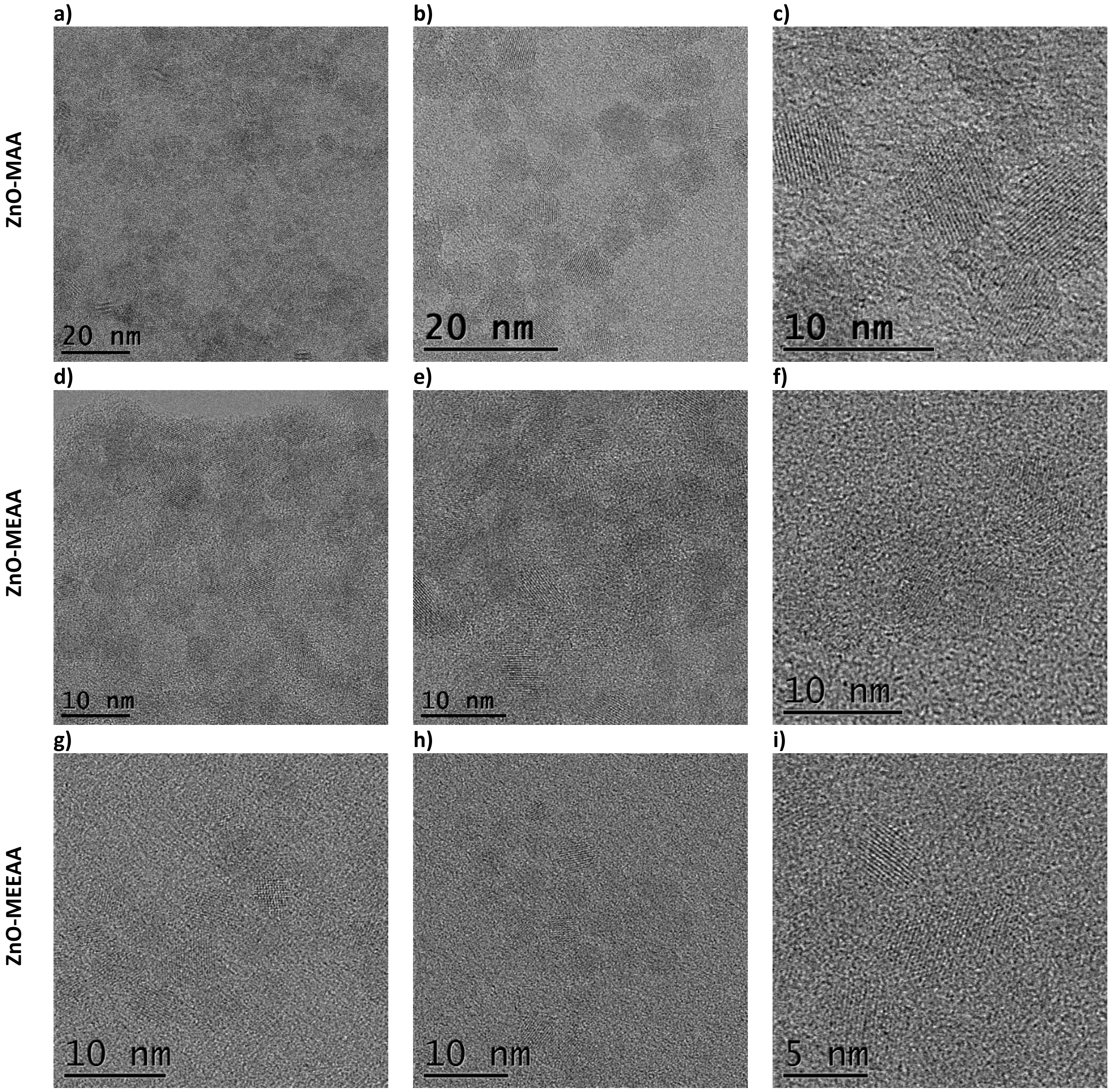

Figure S7. Representative HRTEM images of (a - c) ZnO-MAA, (d - f) ZnO-MEAA, (g - i) ZnOMEEAA NCs.

5. Dispersibility

Table S3. Dispersibility and stability of ZnO-MEAA NCs in various solvents, using color-coded indicators to represent the observed behaviors.

**ZnO-MEAA**	green	green	green	green	green	green	green	green	red	red	green	green	green	red	green	blue	green	green	green	green	green	green
product form	M	P	M	P	M	P	M	P	M	P	M	P	M	P	M	P	M	P	M	P	M	P
solvent	*THF*		*DMSO*		*toluene*		*DCM*		*hexane*		*acetone*		*Et_2O*		*H_2O*		*MeOH*		*EtOH*		*iPrOH*	

Abbreviations: ***M*** *denotes the addition of a post-reaction mixture containing ZnO NCs in THF to a selected solvent in a ratio of 0.1 mL of the mixture to 2 mL of the solvent;* ***P*** *denotes the dissolution of the product in powder form at a concentration of 2 mg per 1 mL of solvent;* ***green*** *indicates a stable, transparent colloidal solution;* ***blue*** *represents a suspension that quickly settles to the bottom; and* ***red*** *red indicates a lack of dispersibility in the given solvent.*

a)

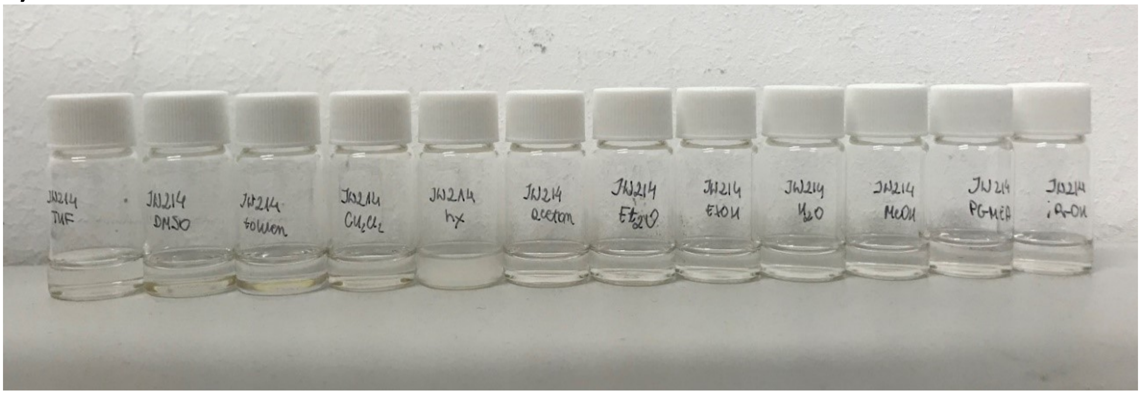

b)

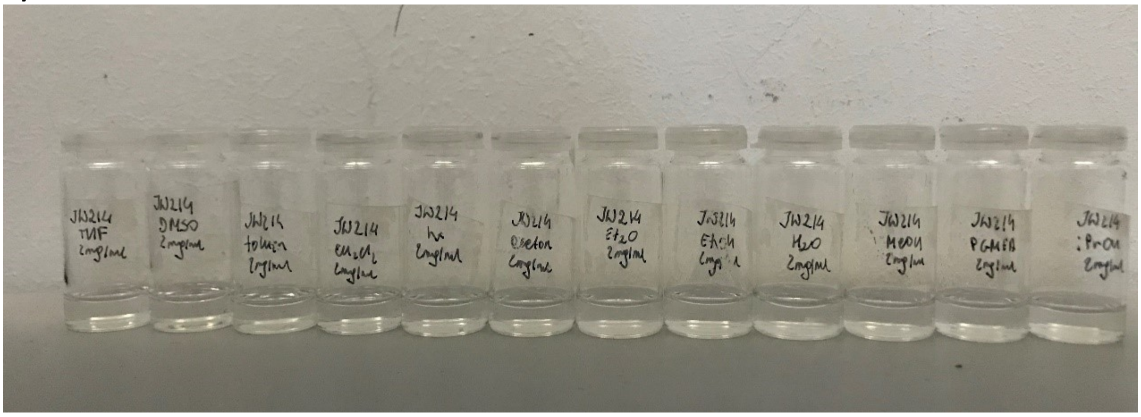

c)

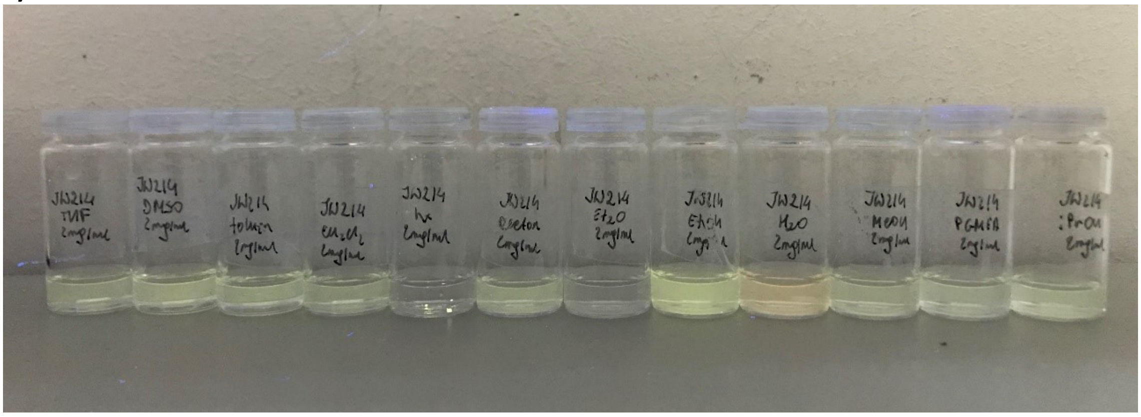

Figure S8. The images present the dispersions of ZnO-MEAA NCs obtained by mixing (a) the post-reaction mixture and (b-c) the product in solid form with selected common organic solvents as well as water. The photographs were taken (a and b) under visible light and (c) UV light (236 nm)."

6. The study of solution properties of colloidal ZnO NCs using the AUC method

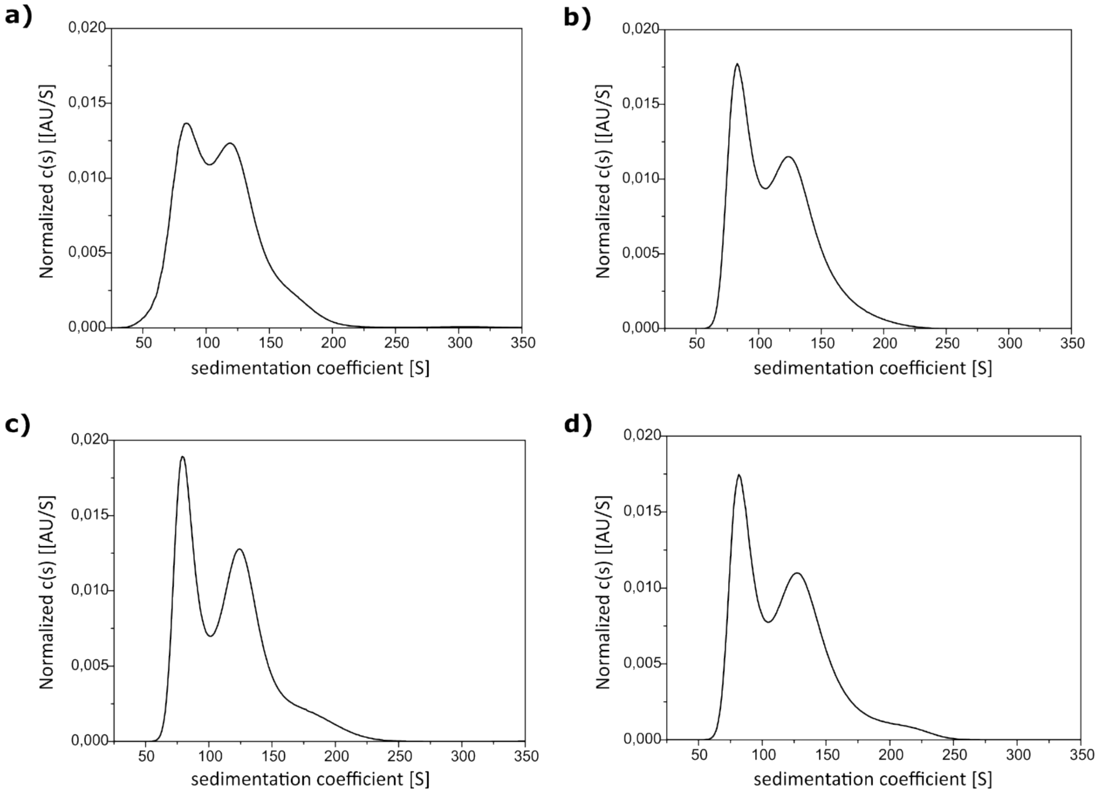

Figure S9. The sedimentation coefficient distribution, c(s), for ZnO-MAA nanocrystals (NCs) in THF was analyzed at 5-day intervals. The results are presented in sequence for the first day (a), sixth day (b), eleventh day (c), and sixteenth day (d) of the experiment.

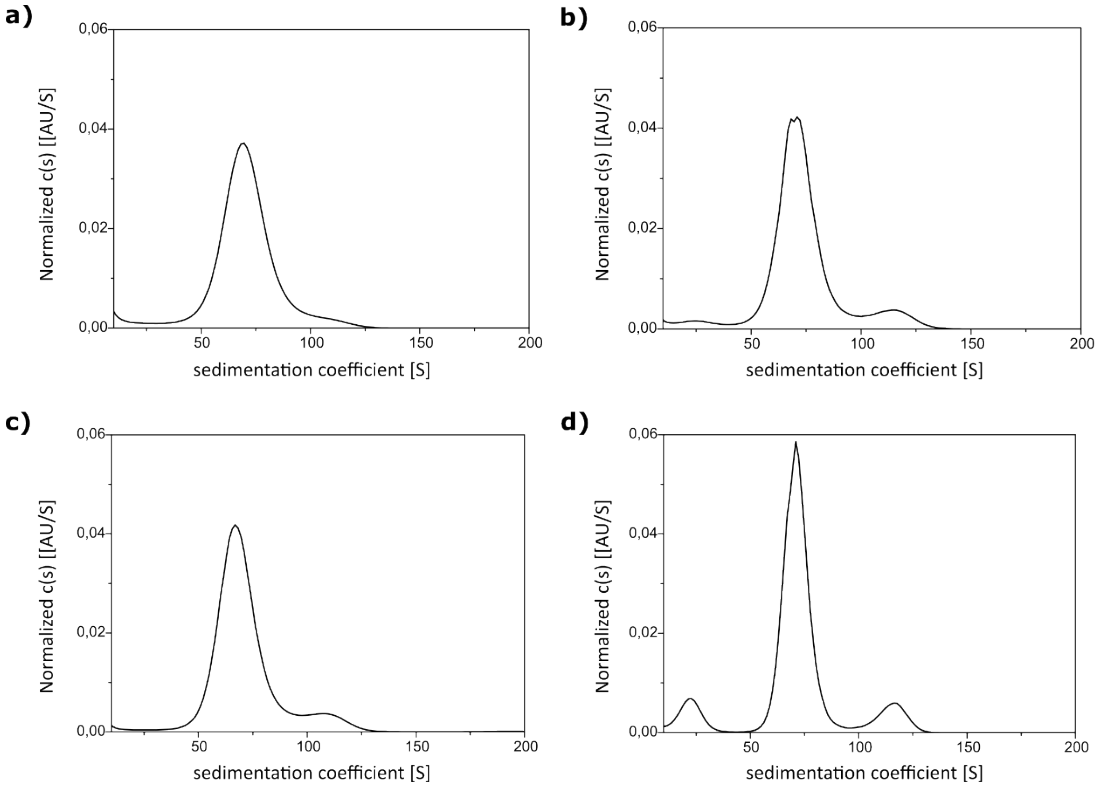

Figure S10. The sedimentation coefficient distribution, c(s), for ZnO-MEAA nanocrystals (NCs) in THF was analyzed at 5-day intervals. The results are presented in sequence for the first day (a), sixth day (b), eleventh day (c), and sixteenth day (d) of the experiment.

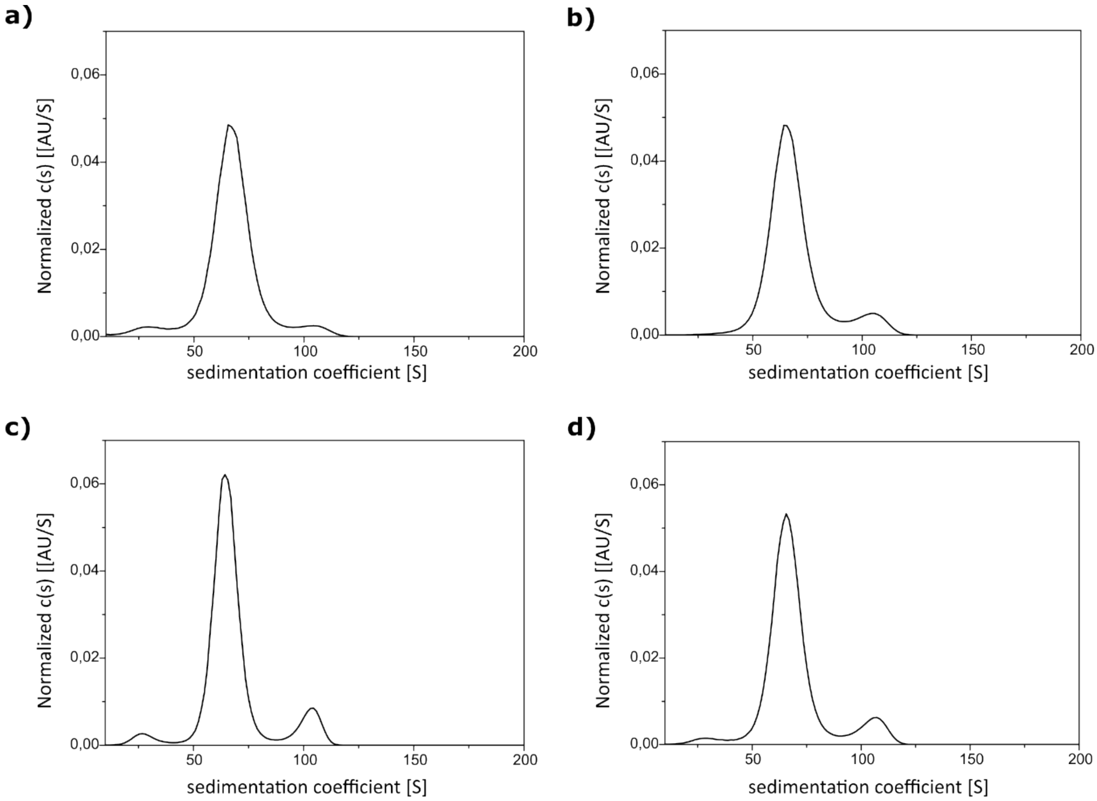

Figure S11. The sedimentation coefficient distribution, c(s), for ZnO-MEEAA nanocrystals (NCs) in THF was analyzed at 5-day intervals. The results are presented in sequence for the first day (a), sixth day (b), eleventh day (c), and sixteenth day (d) of the experiment.

Table S4. Percentage of the signal of individual peaks/sedimentation coefficient zones of ZnO-MAA, ZnO-MEAA, ZnO-MEEAA NCs in relation to the total c(s) signal. Data include 4 centrifugations of each sample over a period of 16 days.

**ZnO-MAA NCs**			
**S range (S)**	**5-60**	**60-180**	**180-260**
1st day,	1.8 %	95.8 %	2.2 %
6th day	0.0 %	97.0 %	2.9 %
11th day	0.1 %	95.0 %	4.9 %
16th day	0.0 %	94.1 %	5.8 %
**ZnO-MEAA NCs**			
**S range (S)**	**10-40**	**40-100**	**100-140**
1st day,	3.3 %	92.7 %	3.9 %
6th day	3.6 %	87.1 %	8.8 %
11th day	1.4 %	91.1 %	7.3 %
16th day	9.0 %	80.9 %	9.9 %
**ZnO-MEEAA NCs**			
**S range (S)**	**7-40**	**40-90**	**90-120**
1st day,	4.5 %	92.4 %	3.2 %
6th day	0.7 %	90.1 %	9.5 %
11th day	3.4 %	86.0 %	10.7 %
16th day	2.5 %	87.4 %	10.1 %
